# Safety and Biocompatibility of a New High-Density Polyethylene-Based Spherical Integrated Porous Orbital Implant: An Experimental Study in Rabbits

**DOI:** 10.1155/2015/904096

**Published:** 2015-11-24

**Authors:** Ivan Fernandez-Bueno, Salvatore Di Lauro, Ivan Alvarez, Jose Carlos Lopez, Maria Teresa Garcia-Gutierrez, Itziar Fernandez, Eva Larra, Jose Carlos Pastor

**Affiliations:** ^1^Instituto Universitario de Oftalmobiologia Aplicada (IOBA), University of Valladolid, 47011 Valladolid, Spain; ^2^Centro en Red de Medicina Regenerativa y Terapia Celular de Castilla y Leon, Valladolid, Spain; ^3^Department of Ophthalmology, Hospital Clinico Universitario, 47005 Valladolid, Spain; ^4^AJL Ophthalmic S.A., 01510 Miñano, Spain; ^5^Networking Research Center on Bioengineering, Biomaterials and Nanomedicine (CIBER-BBN), Valladolid, Spain

## Abstract

*Purpose*. To evaluate clinically and histologically the safety and biocompatibility of a new HDPE-based spherical porous orbital implants in rabbits.* Methods*. MEDPOR (Porex Surgical, Inc., Fairburn, GA, USA), OCULFIT I, and OCULFIT II (AJL Ophthalmic S.A., Vitoria, Spain) implants were implanted in eviscerated rabbis. Animals were randomly divided into 6 groups (*n* = 4 each) according to the 3 implant materials tested and 2 follow-up times of 90 or 180 days. Signs of regional pain and presence of eyelid swelling, conjunctival hyperemia, and amount of exudate were semiquantitatively evaluated. After animals sacrifice, the implants and surrounding ocular tissues were processed for histological staining and polarized light evaluation. Statistical study was performed by ANOVA and Kaplan-Meier analysis.* Results*. No statistically significant differences in regional pain, eyelid swelling, or conjunctival hyperemia were shown between implants and/or time points evaluated. However, amount of exudate differed, with OCULFIT I causing the smallest amount. No remarkable clinical complications were observed. Histological findings were similar in all three types of implants and agree with minor inflammatory response.* Conclusions*. OCULFIT ophthalmic tolerance and biocompatibility in rabbits were comparable to the clinically used MEDPOR. Clinical studies are needed to determine if OCULFIT is superior to the orbital implants commercially available.

## 1. Introduction

The first orbital implants were made of glass, plastic, cartilage, and silicone [1–4]. Although the motility of these implants proved to be excellent, the majority led to necrosis, infection, or exposure and was ultimately removed [5]. Hydroxyapatite and porous polyethylene were first introduced as new implant materials in the 1980s and in the 1990s, respectively [6, 7]. These porous implants have been successfully used for improving prosthetic motility and thus have provided a more natural and cosmetically pleasing look for anophthalmic patients [8].

At present, high-density polyethylene (HDPE) spherical implants, such as MEDPOR (Porex Surgical, Inc., Fairburn, GA, USA), are widely used for implantation into the resulting cavity of eviscerated or enucleated globes. HDPE implants are smooth and malleable, which make the implantation easier [7]. MEDPOR has pores greater than 150 *µ*m, permitting the ingrowth of host vascular and soft tissue. This biointegration reduces the infection rates because it enables immune response to infection and allows delivery of systemically administered antibiotics [9, 10]. Nevertheless, the use of these materials is accompanied by some complications, such as blepharoptosis, eye discharge, implant exposure, conjunctival contracture and/or dehiscence, ectropion, and implant infection and/or extrusion [11]. To reduce complication rates, some changes in implant surface have been made, such as creating a smooth porous anterior surface that helps to reduce implant exposure (MEDPOR SST, Porex Surgical, Inc.) or giving a cone-shaped form to the implant (MEDPOR MCOI, Porex Surgical, Inc.). Although efforts have been made to reduce postoperative complications, reported rates of implant exposure still vary up to 34% [11–15], and it is necessary to remove the implant in up to 29% of the patients [7, 12, 16].

Because of the importance of anophthalmic implants to patients and because of the limitations described above of the existing implants, new materials and implant shape designs are currently under investigation. In this study, we used eviscerated rabbits to evaluate clinically and histologically the safety and biocompatibility of new HDPE-based spherical porous orbital implants (OCULFIT; AJL Ophthalmic S.A., Vitoria, Spain).

## 2. Materials and Methods

### 2.1. Experimental Animals

The use of animals in this study was in accordance with the recommendations of the Association for Research in Vision and Ophthalmology (ARVO) for the Use of Animals in Ophthalmic and Vision Research. It was approved by the Animal Research and Welfare Ethics Committee of the University of Valladolid (Spain) in agreement with European (Council Directive 2010/63/UE) and Spanish regulations (RD 53/2013). Twenty-four (*n* = 24) female rabbits (New Zealand White), weighing between 3.5 and 4.5 kg at implantation time, were used in this study. The animals had normal findings upon complete ophthalmic examinations consisting of slit-lamp biomicroscopy and indirect ophthalmoscopy. Animals were randomly divided into 6 groups (*n* = 4 each) according to 3 implant materials and 2 follow-up times per material (Table 1). HDPE spherical 12 mm diameter implants with smooth porous surface were used. The MEDPOR (*n* = 8; Porex Surgical, Inc.) is a clinically validated implant and served as the control. The OCULFIT I (*n* = 8; AJL Ophthalmic S.A.) is an HDPE-based implant, and the OCULFIT II (*n* = 8; AJL Ophthalmic S.A.) is a similar HDPE-based implant, but it is coated with poly(ethylene glycol) diacrylate (PEGDA) hydrogel. The animals were followed up for 90 (*n* = 12) or 180 days (*n* = 12) after implantation. Animals housing was in accordance with the European regulation with free access to food and water during the experiment.

### 2.2. Surgical Technique

The surgical procedure was performed on the right eye of all rabbits. The animals were anesthetized by an intramuscular injection of ketamine (30 mg/kg; Imalgene 1000, Merial, Lyon, France) and xylazine (6 mg/kg; Rompún 2%, Bayer HealthCare, Kiel, Germany). Pinna and pedal reflexes were used to monitor the level of anesthesia. Prophylactic antibiotic treatment was established with benzylpenicillin procaine/benzathine (7 IU/kg; Shotapen LA, VIRBAC, Carros, France). Analgesia was applied by subcutaneous injection of butorphanol (0.1 mg/kg; Torbugesic Vet, Fort Dodge Animal Health, Fort Dodge, IA, USA). The periorbital area was cleansed by a solution of povidone iodine (5% Betadine; Meda Manufacturing, Bordeaux, France). Topical anesthesia was applied on the right eye prior to the surgical procedure (Colircusí Anestésico Doble; Alcon Cusí S.A., Barcelona, Spain). One milliliter of 1 : 200,000 epinephrine (1 mg/mL; B Braun Medical S.A., Barcelona, Spain) and 2% lidocaine (B Braun Medical S.A.) in phosphate-buffered saline (B Braun Medical S.A.) was given by a retrobulbar injection as a hemostatic agent and to minimize postoperative pain, respectively.

An eyelid speculum was placed to retract the eyelids prior to globe removal. A 360° conjunctival peritomy was performed, and Tenon's capsule was bluntly separated from the underlying sclera. A full-thickness incision around the corneal limbus of the right eye was made using a 15° blade knife (Alcon Laboratories, Inc., Fort Worth, TX, USA), and the entire cornea was removed. After separating the entire uvea from the scleral shell, all intraocular contents were completely removed using an Abadie curette (8 mm; Moria SA, Antony, France). The internal surface of the sclera was wiped with gauze soaked in 96% alcohol to denature any residual uveal pigment. To control hemorrhage, a disposable electrocautery pen (Bovie Medical Corporation, Clearwater, FL, USA) was used at the bleeding points of the scleral shell when necessary. Sclerotomy was performed by four relaxing radial scleral incisions between the rectus muscle insertions. Sterile intraocular 12 mm diameter implants were inserted, and the anterior sclera was closed with 5-0 polyglactin suture (Péters Surgical, Bobigny Cedex, France). The Tenon's capsule and the conjunctiva were closed with 6-0 polyglactin suture (Péters Surgical). Finally, ophthalmic tobramycin (Tobrex Ungüento; Alcon Cusí S.A.) was applied. Fentanyl sustained release patches (25 *µ*g/h; Duragesic Matrix 25, Janssen-Cilag S.A., Madrid, Spain) were used postoperatively to maintain analgesia until 72 h [17, 18].

### 2.3. Clinical Evaluation

After surgery, clinical examinations were performed on days 1, 7, 15, 30, 45, 60, 90, 120, 150, and/or 180. Animals were not sedated for clinical evaluation. Signs of regional tenderness by palpation and presence of eyelid swelling, conjunctival hyperemia, and amount of exudate were evaluated in each rabbit according to Hackett and McDonald irritation and inflammation scoring system [19]. The severity of these clinical signs was investigated and graded as none (0), mild (1), moderate (2), or severe (3) on each day of examination by the same ophthalmologist (SDL). Eye swelling and conjunctival hyperemia were assessed by slit-lamp biomicroscopy (Kowa SL-15; KowaOptimed Inc., CA, USA) in every follow-up time.

### 2.4. Histological Evaluation

The rabbits were anesthetized as previously described and then euthanized with intravenous injection of sodium pentobarbital (200 mg/kg; Dolethal, Vétoquinol, Cedex, France) at 90 or 180 days after implantation. A 5-0 polyglactin suture was placed at the central superior sclera to facilitate sample orientation during tissue processing. Then, the orbital content was exenterated.

The sockets were fixed for at least 24 hours in 10% formalin and then cut through the sagittal plane and processed in an automatic tissue processor (Leica ASP300; Leica Microsystems, Wetzlar, Germany). Two paraffin blocks from each socket were made. After that, multiple 3 *µ*m thick microscope sections at different levels were obtained. Hematoxylin & eosin (HE; Merck KGaA, Darmstadt, Germany) and periodic acid of Schiff (PAS; Merck KGaA) stained slides were examined by standard light microscopy.

Evaluation of the overall inflammatory and tissue responses at the contact surface with the orbital implant was made by an experienced pathologist (JCL). All samples were also examined under polarized light.

### 2.5. Statistical Analysis

Statistical analysis of the clinical parameters was performed using R Statistical Software version 3.1.0 (Foundation for Statistical Computing, Vienna, Austria). The statistical significance level was set at 5%. Given the ordinal nature of clinical parameters, a Kruskal-Wallis one-way nonparametric analysis of variance (ANOVA) was used to compare median values of the three materials at each time point. Homogeneity of variance assumption was checked by the robust Brown-Forsythe Levene-type test using the group medians, implemented in R lawstat package [20]. Pairwise comparisons were performed by Mann-Whitney *U* tests with Bonferroni correction for multiple testing.

For each of the clinical parameters, the number of days until the value reached 0 (stabilization) was also evaluated. Kaplan-Meier survival analysis was applied to estimate the probability that the stabilization time exceeded time* t*. The log-rank test was used to compare the univariate stabilization times of the three implant types. The R survival package was used for this analysis [21].

## 3. Results

### 3.1. Clinical Evaluation

Clinical examinations were performed on days 1, 7, 15, 30, 45, 60, 90, 120, 150, and/or 180. Endpoint times were performed at 90 or 180 days after implantation. However, at 90 days after implantation, one animal in the MEDPOR group and one in the OCULFIT II group were lost to follow-up due to posterior paresis and subsequent ethical sacrifice. Their sockets were removed and submitted for histological processing and evaluation.

### 3.2. Clinical Parameter Follow-Up

**Figure 1:**
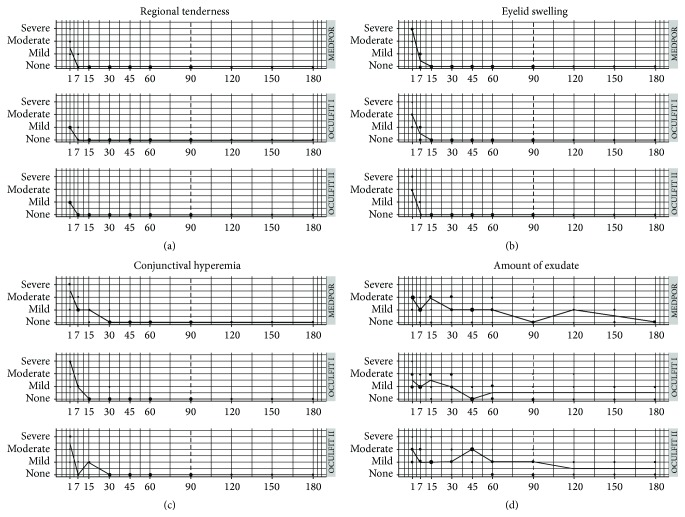
Clinical evaluation follow-up after orbital implantation. The severities of regional tenderness (a), eyelid swelling (b), conjunctival hyperemia (c), and amount of exudate (d) were recorded at each time point after insertion of the MEDPOR, OCULFIT I, and OCULFIT II implants. Clinical signs were graded as none, mild, moderate, or severe according to Hackett and McDonald scoring system [19]. Clinical examinations were performed on days 1, 7, 15, 30, 45, 60, 90, 120, 150, and 180 after implantation. Bubble sizes are proportional to the number of animals.

There were no significant differences in regional tenderness, eyelid swelling, or conjunctival hyperemia among the different orbital implants and/or time points (Figures 1(a)–1(c)). However at 45 days after implantation, the exudate from eyes with OCULFIT II was significantly greater than that from eyes with OCULFIT I (*p* value: 0.0137) (Figure 1(d)). Comparison with MEDPOR was statistically significant at the 10% level (*p* values 0.0968 and 0.0644 for OCULFIT I and OCULFIT II, resp.), and differences in the amount of exudate were statistically different from 0 at 5% level. The amount of exudate in MEDPOR eyes was greater than OCULFIT I eyes (95% confidence interval [CI] MEDPOR, OCULFIT I: [0.04, 1.02]) and lower than OCULFIT II eyes (95% CI MEDPOR, OCULFIT II: [−1.69, −0.16]).

### 3.3. Time to Stabilization

For regional tenderness in all three groups, the average time for the rabbits to reach 0 (none) was 7 days (Figure 1(a)). There were no significant differences among the three survival curves (log-rank test: 1; *p* value: 0.612). For eye swelling in all three groups, the average time to reach 0 (none) edema was 11 days (Figure 1(b)). There were no significant differences among the three survival curves (log-rank test: 0; *p* value: 1). For conjunctival hyperemia, the average time to reach 0 (none) congestion was 15 days for the OCULFIT I group and 30 days for the MEDPOR and the OCULFIT II groups (Figure 1(c)). However, the differences among the three survival curves were not significant (log-rank test: 2.3; *p* value: 0.322). Exudate levels in none of the three groups became stabilized at 0 (none) during the 180-day follow-up (Figure 1(d)). At 90 days, 3 rabbits had 0 amount of exudate in the MEDPOR treated group, 4 in the OCULFIT I group, and only 2 in the OCULFIT II group. The probability of having exudate on day 90 was 0.571, 0.500, and 0.714, respectively, among the groups. There were no significant differences among the three survival curves (log-rank test: 1.1; *p* value: 0.585). At 180 days, the median exudate values were 0 in the MEDPOR group and 0.5 in the OCULFIT I and OCULFIT II groups. For the OCULFIT I group, the median exudate value remained unchanged at 0.5 from 60 days to the end of the experiment. For the OCULFIT II group, the median exudate value remained at 0.5 from 120 days. Neither OCULFIT group reached 0.

### 3.4. Histological Evaluation

**Figure 2:**
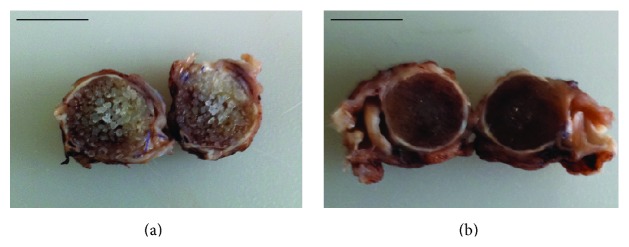
Macroscopic findings at 90 days after orbital implantation. After cutting the eyes through the sagittal plane, the MEDPOR implant (a) appeared as an aggregate of small spherical granules, while OCULFIT I (b), as well as OCULFIT II, was more compact and composed of multiple microgranules. Peripheral ingrowth of host vasculature and soft tissue was present in both materials. Scale bars: 12 mm.

After cutting the sclera with the implants in the sagittal plane, the inner implant materials were exposed. The MEDPOR implant appeared as an aggregate of small spherical granules of about 1 mm diameter (Figure 2(a)). In contrast, both OCULFIT inserts looked more compact and composed of multiple microgranules (Figure 2(b)). On gross examination, peripheral fibrovascularization from orbital tissue was also noted for all three implants.

**Figure 3:**
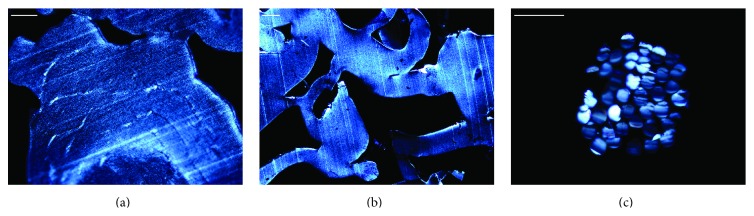
Histological evaluation under polarized light at 90 days after orbital implantation. Orbital implants MEDPOR, OCULFIT I, and OCULFIT II appeared as birefringent intraocular solid material adjacent to the internal surface of the sclera ((a), (b)). Birefringent cylindrical units were present in the peripheral adipose tissue (c). Scale bars: 20 *µ*m ((a), MEDPOR; (c)) and 50 *µ*m ((b), OCULFIT II).

Under polarized light, two types of birefringent materials were identified at 90 and 180 days (Figure 3). All three types of implant specimens appeared as birefringent intraocular solid material adjacent to the internal surface of the sclera (Figures 3(a) and 3(b)). Furthermore, another material composed of birefringent cylindrical units and consistent with surgical suture was present in the peripheral adipose tissue (Figure 3(c)).

**Figure 4:**
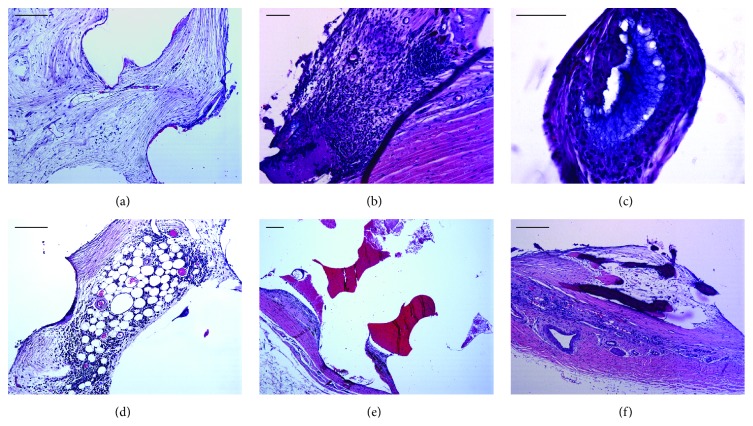
Histological findings in hematoxylin & eosin (HE) stained sections at 180 days after orbital implantation. Loose granulation tissue (a) with an associated foreign body giant cell reaction ((b), (c)) was commonly observed at 90 and 180 days after MEDPOR, OCULFIT I, and OCULFIT II implantation. Metaplastic changes (d), focal intraocular hemorrhage (e), and focal osseous metaplasia (f) were occasionally observed at 180 days after MEDPOR, OCULFIT I, and OCULFIT II implantation, respectively. Scale bars: 50 *µ*m ((a), (d), MEDPOR; (e), OCULFIT I; (f), OCULFIT II) and 20 *µ*m ((b), OCULFIT I; (c), OCULFIT II).

The main histological findings in HE and PAS stained slides were similar in all three types of implants evaluated at both time points. The most common changes included the presence of a loose granulation tissue with an associated foreign body giant cell reaction (Figures 4(a)–4(c)). Also, metaplastic changes (bone marrow metaplasia) were present in two MEDPOR samples (Figure 4(d)), and focal intraocular hemorrhage was present in two OCULFIT I samples (Figure 4(e)). Two OCULFIT II specimens (Figure 4(f)) had focal osseous metaplasia.

## 4. Discussion

The present study described the ophthalmic and histological evaluation of two new HDPE-based spherical integrated porous orbital implants (OCULFIT) in rabbits. Globe removal is a traumatic event for the patient. Cosmetic results are remarkably important to limit the postoperative psychological effects of the patient [22]. In this sense, there were remarkable advancements in orbital implant surgery during the latter part of the 20th century. Spherical integrated porous orbital implants have been widely used throughout the world [9]. However, postoperative complications and implant removal still occur [11–16] and new products have to be studied. Preclinical studies are necessary prior to clinically used approval. In this sense, the socket of rabbits after evisceration of the globe is a widely and currently used model to adequately test intraocular implants [23–25]. In this experimental study in rabbits, we found that, at 90 and 180 days, the tolerance and biocompatibility of the OCULFIT implants was as good as the MEDPOR, an implant in current clinical use.

OCULFIT implants are designed to be implanted into the socket after evisceration of the globes. Different biopolymers have been added to the HDPE to improve flexibility and hydrophilicity of the implants. OCULFIT orbital implants have an interconnected, opened porous structure that allows the ingrowth of host orbital vasculature and soft tissue, which integrates the implant with the host's body. The OCULFIT implants have a smooth anterior surface and a posterior porous surface that helps implant integration and minimizes their exposure. The difference between OCULFIT I and OCULFIT II is that the latter is covered with poly(ethylene glycol) diacrylate (PEGDA) hydrogel, which increases the hydrophilicity and theoretically reduces the integration time of the implants. In the experimental study presented here, both OCULFIT I and OCULFIT II were similar with respect to regional tenderness, eyelid swelling, and conjunctival hyperemia. However, the OCULFIT I caused small amount of exudate during the follow-up period; conjunctival hyperemia was stabilized 15 days before the OCULFIT II; and OCULFIT I had better results regarding the amount of exudate present.

MEDPOR, a polyporous form of polyethylene, is now widely used to compensate for the loss of volume in an anophthalmic socket after globe removal [8, 11]. In addition to its use in anophthalmic socket surgery, MEDPOR is commonly used in craniofacial reconstruction surgery. The porous character of MEDPOR enables fibrovascular proliferation of orbital tissue, reduces the risk of migration, exposure, and extrusion, and minimizes the risk of infection [9, 10]. MEDPOR has a hydrophobic and negatively charged surface that acts as a protective envelope to inhibit the adherence of bacteria and to reduce the postoperative infection rate [26]. This material is also nontoxic, nonallergenic, and highly biocompatible. MEDPOR is currently a very popular orbital implant material and [11], thus, an adequate, clinically validated control to test the tolerance and biocompatibility characteristics of the OCULFIT implants.

In our follow-up clinical evaluations, we found that the three implants tested were similar with respect to regional tenderness, eyelid swelling, and conjunctival hyperemia. However, OCULFIT I caused the smallest amount of exudate during the follow-up period, while MEDPOR induced more and OCULFIT II induced the most exudate. Conjunctival hyperemia was stabilized with OCULFIT I 15 days before the other two materials; and furthermore, it had considerably better results regarding the amount of exudate present. Although the amount of exudate did not become stabilized at 0 during either the 90 days or the 180 days of follow-up periods, the differences between the 0 and 0.5 median exudate values were not clinically relevant. In this case, OCULFIT I became stabilized at 60 days, OCULFIT II at 120 days, and MEDPOR at 180 days. However, there were no significant statistical differences in any case. The presence of continuous exudate during this study may be secondary to implant movement (rubbing) over the sclero-conjunctival surface, as previously described in human patients [22, 27], or remnant tear secretion due to nonremoval of the lacrimal glands. Exudate cultures to detect possible infectious origin were not performed in this study.

Macroscopic evaluation of the implant during tissue processing revealed differences between the MEDPOR and OCULFIT implants regarding the internal structure of the polyethylene granules. This finding may be due to manufacturing differences. However, we observed no differences in microscopic structure when the implants were viewed under polarized light. The birefringent cylindrical units found in the peripheral adipose tissue may correspond to surgical sutures that were used to close the scleral and conjunctival tissues and which did not absorb.

The histological findings were consistent with those previously observed in experimental rabbits [23–25] and in human patients where orbital inflammatory responses to integrated implants, characterized by a foreign body giant cell reaction, have been described [28, 29]. Host tissue growing into an implant does not turn off the foreign body response [7]. During the follow-up period, we found neither implant exposure nor infection clinical signs, which are the most serious complications associated with integrated orbital implants after globe removal [26]. We did find focal intraocular hemorrhage in two OCULFIT I samples. These may have been secondary to implant movement. Metaplastic modifications found in MEDPOR and OCULFIT II specimens were also previously described in anophthalmic sockets with an ocular implant [30].

## 5. Conclusions

In summary, OCULFIT ophthalmic tolerance and biocompatibility in rabbits were comparable to the clinically used MEDPOR orbital implant. Indeed, OCULFIT I had better experimental results. Although OCULFIT II implants induce more exudate in this animal study, the PEGDA hydrogel used to coat them may be loaded with growth factors that can be released in a controlled fashion to reduce the inflammatory response after implantation procedure. In this sense, OCULFIT II implants open a new opportunity to induce rapid integration with the recipient's tissues. Clinical studies are needed to determine conclusively if OCULFIT is superior to the orbital implants commercially available at present.

## Figures and Tables

**Table 1:** Animal distribution and follow-up time for each orbital implant type.

Implants type	Number of animals (*n*)	Follow-up (days)
MEDPOR (Porex Surgical, Inc., Fairburn, GA, USA)	4	90
	4	180

OCULFIT I (AJL Ophthalmic S.A., Vitoria, Spain)	4	90
	4	180

OCULFIT II (AJL Ophthalmic S.A., Vitoria, Spain)	4	90
	4	180
